# The Genetic Profile and Serum Level of IL-8 Are Associated with Chronic Hepatitis B and C Virus Infection

**DOI:** 10.3390/biom11111664

**Published:** 2021-11-10

**Authors:** Ednelza da Silva Graça Amoras, William Botelho de Brito, Maria Alice Freitas Queiroz, Simone Regina Souza da Silva Conde, Izaura Maria Vieira Cayres Vallinoto, Ricardo Ishak, Antonio Carlos Rosário Vallinoto

**Affiliations:** 1 Laboratory of Virology, Institute of Biological Sciences, Federal University of Pará (Universidade Federal do Pará—UFPA), Belém 66075-110, Brazil; ednelza@ufpa.br (E.d.S.G.A.); britowilliam2012@gmail.com (W.B.d.B.); alicefarma@hotmail.com (M.A.F.Q.); ivallinoto@ufpa.br (I.M.V.C.V.); rishak@ufpa.br (R.I.); 2 João de Barros Barreto Hospital, Federal University of Pará (Universidade Federal do Pará—UFPA), Belém 66073-000, Brazil; sconde@ufpa.br; 3 Institute of Health Sciences, School of Medicine, Federal University of Pará (Universidade Federal do Pará—UFPA), Belém 66075-110, Brazil

**Keywords:** HBV, HCV, IL-8, polymorphism, plasma levels

## Abstract

The present study evaluated the *IL8*-251 A/T polymorphism in samples from 74 patients with chronic hepatitis B (HBV), 100 patients with chronic hepatitis C (HCV), and 300 healthy donors (CG). The correlations of this polymorphism with plasma IL-8 and disease stage were calculated. Polymorphisms were identified by real-time PCR. IL-8 was measured by enzyme-linked immunosorbent assay. The *IL8*-251 A/T genotype was not associated with susceptibility to infection by HBV or HCV. The wild-type allele (A) was associated with higher levels of inflammation (*p* = 0.0464) and fibrosis scores (*p* = 0.0016) in the HBV group, representing an increased risk for increased inflammatory activity (*OR* = 1.84; *p* = 0.0464) and for high fibrosis scores (*OR* = 2.63; *p* = 0.0016). Viral load was higher in HBV patients with polymorphic genotypes (TA and TT) at the *IL8*-251 A/T polymorphism than in those with the wild-type genotype (*p* = 0.0272 and *p* = 0.0464, respectively). Plasma IL-8 was higher among patients infected with HBV or HCV than in the control group (*p* = 0.0445 and *p* = 0.0001, respectively). The polymorphic genotype was associated with lower IL-8 than the wild-type genotype in the HBV group (*p* = 0.0239) and the HCV group (*p* = 0.0372). The wild-type genotype for *IL8*-251 A/T and high IL-8 were associated with a worse prognosis for infections; therefore, they may contribute to viral persistence and the development of more severe forms of chronic viral liver diseases.

## 1. Introduction

The hepatic lesion triggered by the hepatitis B and C viruses is mediated mainly by host immune responses to viral proteins expressed in infected hepatocytes and, to a lesser extent, by direct cytopathic effects of viruses [1]. Therefore, variations in the natural course of infections and the severity of liver inflammation are determined by interactions between viruses and host factors, which result in a broad spectrum of clinical manifestations of these infections, ranging from mild and asymptomatic forms to more severe forms of liver disease, such as hepatocarcinogenesis [2,3].

Although the immune system has adapted to reduce the liver damage caused by immune responses to infectious agents, the infection caused by the hepatitis B virus (HBV) and the hepatitis C virus (HCV) can lead to hepatocellular death and, consequently, liver injury [1].

In chronic viral hepatitis, inflammation is a persistent immune response. In this condition, inflammation and tissue remodeling and repair processes occur simultaneously inducing fibrosis, which is initially perceived as a physiological mechanism capable of limiting the extent of the inflammatory process [4]. However, with persistent injury, this repair process becomes pathological, characterized by extracellular matrix (ECM) deposition, parenchymal cell death, and angiogenesis, together with tissue remodeling [4]. In chronic hepatitis, activated hepatic stellate cells (HSCs) become myofibroblasts and play a dominant role in fibrosis by producing large amounts of collagen, while the upregulation of tissue inhibitor of metalloproteinases-1 (TIMP-1) contributes to collagen deposition by inhibiting ECM resolution [5]. The persistent production of growth factors for HSCs, fibrogenic cytokines, and chemokines by various types of liver cells is involved in fibrogenesis and chronic inflammation [5]. This scar matrix typically accumulates very slowly, but once cirrhosis is established, the potential to reverse this process is diminished and complications develop [3]. Knowledge of the stage of liver fibrosis provides essential information for the prognosis and helps to determine whether antiviral therapy is necessary, in addition to allowing for differential diagnosis with other liver diseases [6].

Cytokines play an important role in regulating the inflammatory process of the liver through the activation of effector cells. However, differences in cytokine production may lead to a dysregulated inflammatory process [7]. The cytokine interleukin (IL)-8, also known as C-X-C motif ligand 8 (CXCL8), is a proinflammatory chemokine that acts as a chemoattractant of leukocytes. It is secreted by several cells—such as macrophages, neutrophils, and epithelial cells—to promote immune infiltration and angiogenesis and mediate the activation and migration of peripheral blood neutrophils to tissues, thus increasing local inflammation [8,9].

The *IL8* gene harbors several genetic variations, including the *IL8*-251 A/T (rs4073) polymorphism, which has been associated with changes in transcriptional activity and reduced serum IL-8 [10]. Clinical studies have linked high serum IL-8 with poor prognosis of diseases because chemokine expression activates the NF-κβ (nuclear factor-κβ) pathway, which exacerbates the inflammatory cycle [11]. High serum IL-8 has also been associated with chronic viral hepatitis and hepatic crisis [12,13].

The objective of this study was to investigate the association of the *IL8*-251 A/T polymorphism with changes in the plasma level of IL-8, which might influence the progression of chronic diseases caused by HBV and HCV.

## 2. Materials and Methods

### 2.1. Study Design and Population Evaluated

This cross-sectional study was conducted at the liver disease outpatient clinic of the Hospital Universitário João de Barros Barreto (João de Barros Barreto University Hospital) and the Hospital Santa Casa de Misericórdia (Santa Casa de Misericórdia Foundation Hospital) in the state of Pará, Brazil, where consecutive cases of patients with chronic HBV and HCV who had not received antiviral treatment were selected at the time of diagnosis.

All selected patients were clinically evaluated, received complementary investigation, and were divided into two groups. The first group comprised 74 patients with chronic hepatitis B, who were characterized by clinical changes, liver tests, positive HBsAg, and positive or negative HBeAg. The second group consisted of 100 patients with chronic hepatitis C, who were characterized by clinical abnormalities and positive HCV-RNA in liver tests. A control group composed of 300 volunteer blood donors of the Hemotherapy and Hematology Center Foundation of Pará who were seronegative for HBV, HCV, human immunodeficiency virus (HIV), human T-lymphotropic virus, Chagas disease, and syphilis, was used to compare the frequencies of the polymorphism.

The adopted inclusion criteria included individuals aged 18 years and older, from both sexes, carrying HBsAg for more than 6 months, with positive HCV-RNA, and with or without high alanine aminotransferase (ALT) persistence. Patients coinfected with hepatitis delta virus (HDV) or HIV, patients who received specific anti-HBV and anti-HCV antiviral therapy, and patients with hepatocellular carcinoma (HCC) diagnosis were excluded from the study.

### 2.2. Genotyping of IL8 A/T (rs4073)

DNA was extracted from peripheral-blood leukocytes using the Puregene kit (Gentra Systems, Minneapolis, MN, USA) according to the manufacturer’s protocol, which included cell lysis, protein precipitation, DNA precipitation, and rehydration.

The *IL8*-251 A/T polymorphism was genotyped by real-time polymerase chain reaction (qPCR) in the StepOne PLUS Sequence Detector (Applied Biosystems, Foster City, CA, USA). The assay used for genotyping the *IL-8*-251 A/T polymorphism was the predesigned and commercially available TaqMan (C_11748116_10) assay (Thermo Fisher, Carlsbad, CA, USA). This assay consists of a pair of primers and two probes, VIC (for identification of the A allele), and FAM (for identification of the T allele). The primer and probe sequences are not available from the company. For each reaction, TaqMan Universal PCR Master Mix (1×), TaqMan Assay (20×), and 20 μL of DNA were used to produce a final reaction volume of 10 mL. In the amplification reactions, the following temperature cycling was used: 60 °C for 30 s, 95 °C for 10 min, and 50 cycles of 92 °C for 30 s and 60 °C for 1 min and 30 s.

### 2.3. Laboratory Data

Information on the serology of viral liver diseases, levels of liver enzymes, and plasma viral loads were obtained from updated clinical records. These data were organized in a worksheet with restricted access and were used only to obtain information related to the study objectives.

### 2.4. Histopathological Analysis

Liver biopsy specimens were taken only from patients with medical indication for the investigation of liver parenchyma abnormalities and staging the degree of fibrosis as a clinical parameter for the indication of antiviral treatment or not, within the clinical care protocol. Hepatic biopsies were performed by a medical professional at each hospital according to the established medical protocol. Each sample was examined at the Pathology Anatomy Service of the Federal University of Pará (UFPA) according to the routine protocols of the service.

The histopathological diagnosis followed the French METAVIR classification [6], which classifies the activity of portal and periportal inflammatory infiltrate with a score from 0 to 3 (A0–A3) with A0–A1 indicating inflammation absent to mild and A2–A3 indicating moderate to severe inflammation. Structural alterations of the liver parenchyma (degree of fibrosis) were classified from 0 to 4 (F0–F4), with F0–F1 indicating absent to light liver fibrosis, F2 indicating moderate liver fibrosis, and F3–F4 indicating advanced liver fibrosis to cirrhosis. All data on the histopathological profile were obtained from the patients’ medical records.

### 2.5. Quantification of Plasma IL-8

Plasma IL-8 was measured by enzyme-linked immunosorbent assay (ELISA) immunoenzymatic test (ELISA Kit Human IL-8 Ultrasensitive, ThermoFisher, Camarillo, CA, USA). This method used specific monoclonal antibodies to detect the cytokine and was performed according to the instructions provided by the manufacturer. The measurement of plasma IL-8 was performed for a different number in each study group: HBV (n = 55), HCV (n = 66), and control group (n = 55).

### 2.6. Statistical Analysis

The distribution of genotypes was evaluated for its adherence to Hardy–Weinberg equilibrium. The allelic and genotypic frequencies were estimated by direct counting. These frequencies were compared between the groups by the chi-squared test and the G test. The plasma levels of IL-8 were compared between genotypes by the Kruskal–Wallis test and between groups by the Mann–Whitney test. The correlation was evaluated with Spearman’s test. The statistical analyses were performed with the software BioEstat v5.0 [14] with an adopted significance level of *p* < 0.05.

### 2.7. Plasma Viral Load 

Plasma viral load was measured by real-time PCR using an Abbott RealTime HCV Amplification Reagent Kit (Abbott Park, IL, USA) at the Central Laboratory of the State of Pará (LACEN-PA). HBV viral load quantifications are presented as copies/mL and log_10_ value. The lowest detection level of HBV load was 1.08 log UI/mL, and the highest detection level was 9.00 log UI/mL. HCV viral load quantifications are presented as copies/mL and log_10_ value. The lowest detection level of HCV load was 1.08 log UI/mL, and the highest detection level was 8.00 log UI/mL.

## 3. Results

The majority of the patients in the HBV group were male (n = 46; 62.2%). The median ALT, aspartate aminotransferase (AST), and gamma-glutamyl transferase (GGT) liver enzymes were 29 IU/L, 29 IU/L, and 34.5 IU/L, respectively, and the log_10_ (viral load) was 2.8. In the HCV group, most of the patients were male (n = 51; 51%), with median ALT, AST, and GGT levels of 58 IU/L, 59 IU/L, and 67 IU/L, respectively. The log_10_ (viral load) was 5.4 (Table 1).

According to the METAVIR classification (Table 1), the majority of patients with HBV were classified as having an absent to moderate degree of fibrosis (F0–F2) (n = 62; 83.8%) and an absent or mild degree of inflammatory activity (A0–A1) (n = 61; 93.8%). In the HCV group, the highest proportion of patients were F0–F2 (n = 69; 69%) and A0–A1 (n = 50; 54.9%).

Inflammatory activity was assessed in only 65 HBV patients and 91 HCV patients because nine patients from each group were diagnosed with liver cirrhosis through imaging exams and therefore did not meet the medical indication criteria for biopsy.

The Hardy–Weinberg equilibrium analysis showed that the genotypic frequencies of *IL8*-251 A/T were in equilibrium in each group. The evaluation of *IL8*-251 A/T demonstrated that there were no statistically significant allele or genotype differences between groups (Table 2).

The analyses of inflammatory activity and fibrosis score when related to the *IL-8*-251 A/T polymorphism revealed that, in the HBV group, the polymorphic allele (T) was more frequent in patients with lower inflammatory activity (A0–A1) and a low degree of fibrosis (F0–F2), whereas the wild-type allele (A) was more frequent in individuals with a high degree of inflammation and in individuals with a high degree of fibrosis (*p* = 0.0464 and *p* = 0.0016; Table 3). The wild-type allele was associated with increased risk of more severe forms of inflammatory activity (*OR* = 1.84; *p* = 0.0464) and fibrosis (*OR* = 2.63; *p* = 0.0016).

**Figure 1:**
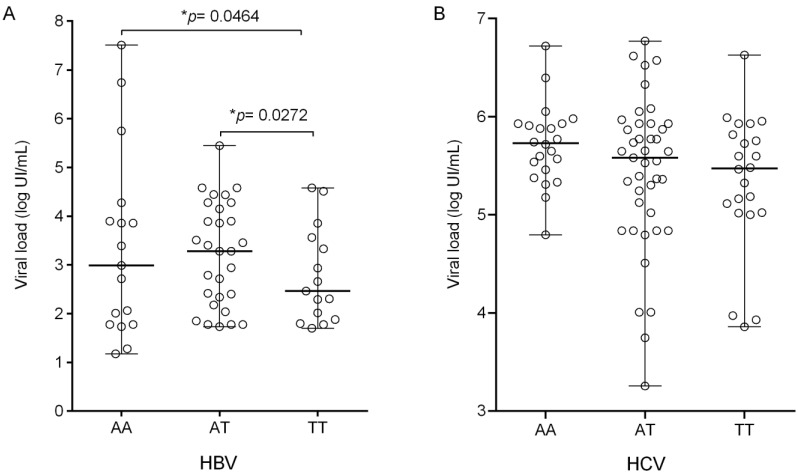
Viral load in the different genotypes of the *IL8*-251 A/T polymorphism in patients with (**A**) HBV: AA (n = 17; median: 3.00; IQR: 2.30), AT (n = 41; median: 3.28, IQR: 1.95), TT (n = 16; median: 2.56, IQR: 1.68) and (**B**) HCV: AA (n = 20; median: 5.58, IQR: 0.49), AT (n = 49; median: 5.72, IQR: 1.01) and TT (n = 31; median: 5.47, IQR: 0.83). * Kruskal–Wallis test.

Patients with genotypes with the wild-type allele (AA and AT) had a higher median HBV viral load than TT patients, with statistical significance (*p* = 0.0272 and *p* = 0.0464, respectively; Figure 1A). The HCV viral load level did not differ between carriers of different genotypes, although the lower viral load levels were grouped into the polymorphic genotype TT (Figure 1B).

**Figure 2:**
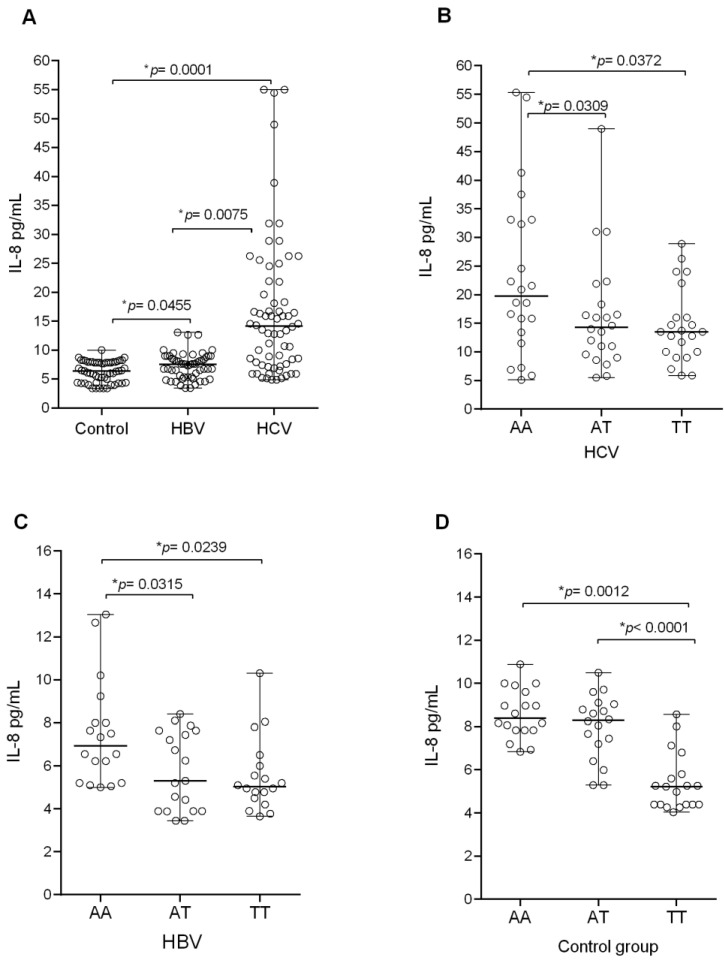
Plasma IL-8 levels according to the genotypes at the *IL8*-251 A/T polymorphism. (**A**) The investigated groups: Control group (n = 55; median: 6.40; IQR: 3.57), HBV (n = 55; median: 7.55; IQR: 3.66), and HCV (n = 66; median: 14.15; IQR: 14.39). (**B**) HCV: AA (n = 22; median: 19.75; IQR: 20.95), AT (n = 22; median: 14.30, IQR: 9.78), and TT (n = 22; median: 13.5; IQR: 7.75). (**C**) HBV: AA (n = 18; median: 6.94; IQR: 3.47), AT (n = 19; median: 5.30, IQR: 3.75), and TT (n = 18; median: 5.02; IQR: 1.70). (**D**) Control group: AA (n = 18; median: 8.38; IQR: 1.83), AT (n= 18; median: 8.28, IQR: 2.04), and TT (n = 19; median: 5.22; IQR 1.41). * Kruskal–Wallis test.

Among the groups studied, the median plasma IL-8 levels were higher among patients infected with HCV, followed by HBV (*p* < 0.001) and the control group, such that when compared with HBV and HCV the differences were significant (*p* = 0.0445 and *p* = 0.0075, respectively; Figure 2A). The polymorphic genotype TT was associated with lower levels of IL-8 and the wild genotype AA with higher levels of cytokine in all of the investigated groups (Figure 2B–D). In the polymorphic genotypes AT and TT, IL-8 levels were lower in all groups when compared with the AA genotype, and the comparison between these genotypes indicated significance. In the HCV group (AA vs. AT: *p* = 0.0309; AA vs. TT: *p* = 0.0372), in the HBV group (AA vs. AT: *p* = 0.0315; AA vs. TT: *p* = 0.0239), and in the control group (AA vs. TT: *p* = 0.0012; AT vs. TT: *p* < 0.0001).

**Figure 3:**
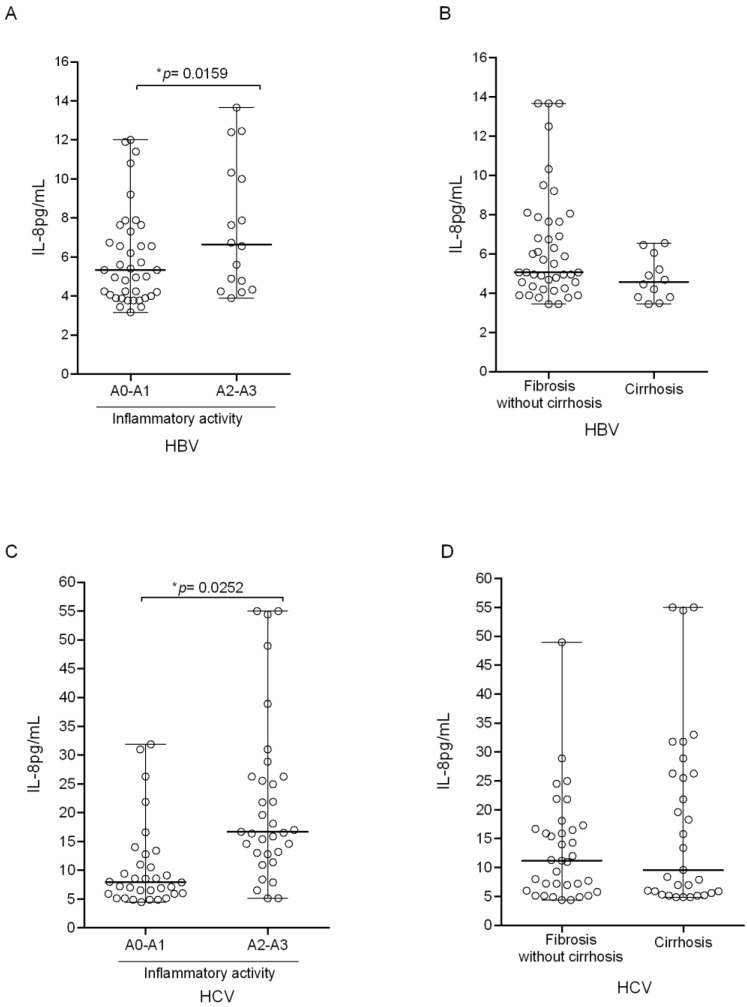
Plasma IL-8 levels according to the fibrosis stages and necroinflammatory activity in the liver tissue of the patients. (**A**) Relationship between IL-8 and necroinflammatory activity in HBV carriers: A0–A1 (n = 39, median: 5.3, IQR: 3.3); A2–A3 (n = 16, median: 6.8, IQR: 5.8). (**B**) Relationship between IL-8 and cirrhosis in HBV carriers: Fibrosis without cirrhosis (n = 43, median: 5.0, IQR: 3.3); Cirrhosis (n = 12, median: 4.5, IQR: 3.3). (**C**) Relationship between IL-8 and necroinflammatory activity in HCV carriers: A0–A1 (n = 33 median: 7.9, IQR 5.9); A2–A3 (n = 33 median: 16.8, IQR: 13.3). (**D**) Relationship between IL-8 and cirrhosis among HCV carriers: Fibrosis without cirrhosis (n = 35 median: 11.2, IQR: 10.7); Cirrhosis (n = 31 median: 9.6, IQR: 20.6). * Mann–Whitney test.

The analysis of cytokine levels in relation to the stages of fibrosis and necroinflammatory activity in the liver tissue demonstrated similarity between HBV and HCV infections. Patients with moderate to severe necroinflammatory activity (A2–A3) had significantly higher IL-8 levels than A0–A1 in both groups: HBV (*p* = 0.0159; Figure 3A) and HCV (*p* = 0.0252; Figure 3C). In the fibrosis stages, IL-8 levels showed no difference in either HCV or HCV (Figure 3B,D).

**Figure 4:**
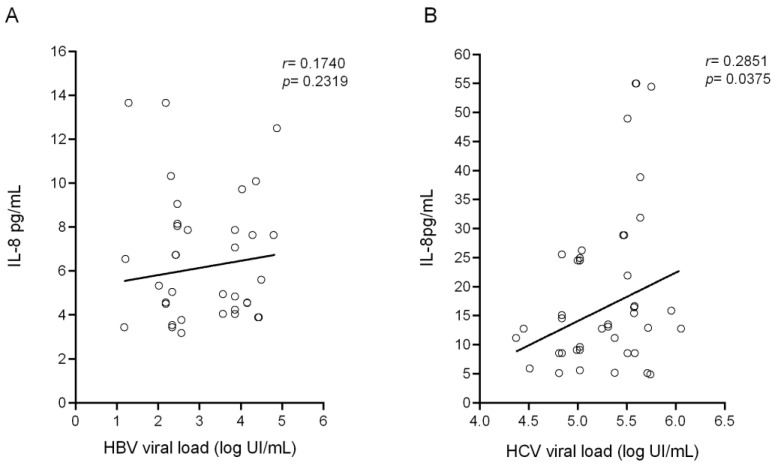
Spearman’s correlation between plasma IL-8 levels and viral load in groups with chronic HBV (**A**) and HCV (**B**) infection.

Figure 4 demonstrates that there was a nonsignificant positive correlation between plasma IL-8 and HBV viral load (Figure 4A), but for HCV the correlation was positive and significant (*p* = 0.0375; Figure 4B).

## 4. Discussion

Genetic variations in the genes encoding important cytokines have been correlated with the susceptibility to or evolution of several viral infections [15,16,17]. IL-8 is a proinflammatory chemokine whose production is induced by certain viral infections and is involved in the induction of neutrophil chemotaxis, angiogenesis, and hematopoiesis [9]. *IL8* gene polymorphisms have been evaluated in HBV and HCV infection [18,19].

In the present study, although the frequency of the polymorphic genotype (TT) at the *IL8*-251 A/T polymorphism was lowest in the chronic HBV carriers group, this difference was not significant between the HCV group and the control group. In a previous study, a higher frequency of heterozygous polymorphic genotypes was observed in patients infected with HBV or HCV in Turkey, which could play an important role in the chronicity of infections [19]. The analysis of these different results may be related to the ethnic differences between the populations evaluated. Patients included in this study were from the Amazon region of Brazil with different genetic backgrounds, including whites, Africans, and indigenous peoples [20]. Thus, genetic analysis of the variant *IL8*-251 A/T alone is sufficient to predict susceptibility to chronic infection by the viruses investigated. The analysis of other factors is important to evaluate the complexity of the chronicity of hepatitis B and hepatitis C.

The analysis of histopathological markers in relation to the *IL8*-251 A/T polymorphism showed that the higher frequency of the wild allele (A) was related to high levels of inflammation (A2–A3) and fibrosis scores (F3–F4) in HBV. This allele was associated with an almost 2-fold increased risk of higher levels of inflammatory activity and an almost 3-fold increased risk of higher fibrosis scores. The wild-type allele for the *IL8*-251 A/T polymorphism is associated with high IL-8 expression, which causes an increase in neutrophil attraction and local inflammatory processes, leading to the development of a fibrotic process, which may progress to cirrhosis [8,10]. In contrast, an evaluation of Chinese patients with chronic hepatitis B revealed that the wild-type genotype was associated with a decreased risk of liver cirrhosis [18]. This discrepancy might be related to the different groups used for comparison to verify the association: the present study evaluated the differences in the levels of inflammation and fibrosis in patients who had chronic HBV infection, while Qin et al. (2012) compared HBV patients with cirrhosis with a healthy control group [18].

The viral load of HBV was higher in individuals with at least one wild-type allele at the *IL8*-251/T polymorphism. Few studies have investigated the *IL8*-251 A/T polymorphism in HBV infection [18,19], and none have assessed its influence on the viral load. In the HCV group, the viral load was similar between the three genotypes. These differences in results may be related to the different characteristics between HBV and HCV in terms of the immune response. For example, HBV has a low mutation rate, whereas HCV has a high mutation rate (there are more than 50 subtypes), which seems to give it a greater ability for immune escape [1].

The comparison of plasma IL-8 levels between the investigated groups revealed that patients with HBV and HCV had higher levels than the control. Other studies have also observed higher IL-8 in patients with chronic hepatitis B or C [2,3,4,5,6,7,8,9,10,11,12,13,14,15,16,17,18,19,20,21,22,23], corroborating the role of this cytokine in the immunopathogenesis of these viral infections and the worsening of the hepatic lesion [12,13].

Levels of IL-8 appeared to be influenced by the different genotypes of the *IL8*-251 A/T polymorphism in all groups investigated. Patients with chronic viral hepatitis and those with polymorphic genotypes (TA and TT) exhibited significantly lower cytokine levels, similar to previous studies on respiratory syncytial virus infection [10,24]. These results are important because they show the relationship between this polymorphism and a cytokine level both in healthy individuals and in patients chronically infected with HBV or HCV. The results also suggest that, among patients, carriers of different genotypes may progress to the most severe form of disease differently. As this is the first study that evaluated IL-8 level in relation to the *IL8*-251 A/T gene polymorphism in chronic viral hepatitis, other similar studies in different ethnic groups would be helpful to further define the roles of IL-8 and this polymorphism in chronic HBV and HCV infection.

We observed an association of higher plasma IL-8 with higher levels of inflammatory activity (A2–A3) in HBV and HCV. Yang et al. (2014) reported that patients with chronic hepatitis B had higher serum IL-8 and higher liver tissue IL-8 mRNA, and IL-8 expression increased with the severity of liver inflammation and fibrosis stage [13]. In one study of HCV-infected patients, serum IL-8 increased as the severity of chronic hepatitis C progressed to the development of hepatocarcinoma; therefore, it can be used as a prognostic factor for the development of this cancer [25]. Increased IL-8 was related to severe liver damage, and its levels decreased when these conditions improved in HBV [26]. Additionally, this cytokine is considered to be one of those responsible for maintaining the inflammatory environment in HBV infection and was associated with HCC interfering with antitumor immunity [27].

The correlation of viral load with IL-8 level showed a weak relationship in the HBV group, perhaps attributed to the sample size in IL-8 dosages in the HBV group, and a strong positive correlation in the HCV group, suggesting that high IL-8 favors the replication of the virus in chronic hepatitis C [22,28]. IL-8 has been associated with HCV replication; in acute infection, it is produced as a consequence of the innate immune response against the virus, and in chronic infection, the induction of high IL-8 caused by viral persistence could induce hepatic pathology [29]. The NS5A protein of HCV has been associated with the upregulation of IL-8, promoting the inhibition of the activation of interferons and favoring the persistence of viral infection [22].

In our results, it is likely that the high levels of IL-8, in addition to the association with the wild genotype *IL8*-251 AA, also influenced the pathogenesis of chronic viral infection, corroborating previous studies, such as the case of HCV in which regulatory cells (Tregs) Foxp3^+^ CD4^+^ were identified as an additional intrahepatic source of IL-8 in chronic hepatitis. IL-8-producing Tregs stimulate HSC by local release of IL-8, leading to fibrogenesis. This IL-8 secretion was related to chronic active HCV infection, rather than a general feature of intrahepatic Tregs. Thus, IL-8-producing Tregs have the potential to simultaneously suppress inflammatory activity through regulatory mechanisms and induce fibrogenesis [30]. Furthermore, the nonspecific inflammatory infiltration of CD8^+^ T cells into the liver, responsible for liver damage in hepatitis C, occurs through the interaction between secreted intrahepatic chemokines and chemokine receptors expressed on CD8^+^ T cells [31].

In chronic HBV infection, IL-8 control also appears to be complex and multifaceted, with the involvement of different signaling pathways, as shown by studies in which IL-8 inhibition increased the antiviral activity of IFN-α against HBV, indicating that: HBV activates the expression of the IL-8 gene aiming at the epigenetic regulation of the IL-8 promoter; IL-8 can contribute to reducing the sensitivity of HBV to IFN-α [32]; chronic infected flares of liver inflammation (spontaneous or induced by antiviral withdrawal) are preceded by a parallel increase in interleukin-8 (IL-8) production and serum HBV DNA levels; IL-8 can synergize with interferon-alpha (IFN-α) to activate NK cells [33,34]; and HBV-specific T cells that mature in the intrahepatic inflammatory environment can produce IL-8, which in turn can contribute to the development of liver damage through the recruitment of granulocytes [26,35].

Thus, IL-8 pathways are involved in the pathogenesis of chronic viral liver disease. In this context, mechanisms that increase the levels of this cytokine, such as the wild genotype of the *IL8*-251 A/T polymorphism and/or immune response mechanisms, may favor the development of the most severe forms of the disease, further supporting the possible role of IL-8 in inflammation and fibrogenesis. Otherwise, it is fair to think that inhibiting IL-8 production or activity would be an alternative to suppressing viral activity, reducing or blocking liver inflammation, and improving IFN-α antiviral action with effective immunotherapeutic approaches against HBV.

With regard to limitations in our study, we mention the difficulty in selecting chronic HBV and HCV carriers before antiviral therapy, in addition to this population being part of a public health service in the State, with its own flow and demand, which, for ethical reasons of the service, limited our access to some personal information and prevented us from obtaining other biological samples from patients.

## 5. Conclusions

In summary, the wild-type genotype at *IL8*-251 A/T and a high level of IL-8 were associated with a worse prognosis of HBV and HCV infections. This genotype and a high IL-8 level may contribute to viral persistence and the development of more severe forms of chronic viral liver diseases.

## Figures and Tables

**Table 1:** Clinical, laboratory, and histopathological data of study participants with chronic HBV and HCV.

Variables	HBV (n = 74)	HCV (n = 100)
Sex, F/M (%)	28/46	49/51
ALT (IU/L), median/IQR	29/37	58/59
AST (IU/L), median/IQR	29/30	59/54.5
GGT (IU/L), median/IQR	34.5/38.75	67/90.5
Viral load (log_10_), median/IQR	2.8/1.7	5.4/0.7
Fibrosis score		
0 to 2	62 (83.8%)	69 (69%)
3 to 4	12 (16.2%)	31 (31%)
Inflammatory Activity	*	**
0 to 1	39 (75.4%)	50 (54.9%)
2 to 3	16 (24.6%)	41 (45.1%)

ALT: alanine aminotransferase (reference value: 16–40 IU/L); AST: aspartate aminotransferase (reference value: 08–54 IU/L); GGT: gamma-glutamyl transferase (reference value: 08–63 IU/L). Fibrosis score METAVIR: 0: absence of septa; 1: portal fibrosis without septa; 2: portal fibrosis with rare septa; 3: numerous septa but without cirrhosis; 4: cirrhosis. Inflammatory activity: 0: absence of activity; 1: minimum activity; 2: moderate activity; 3: intense activity. IQR: interquartile range. Inflammatory activity: * n = 65; ** n = 91.

**Table 2:** Allele and genotype frequencies at the *IL8*-251 A/T polymorphism in the chronic hepatitis B, chronic hepatitis C, and control groups.

Genotypic and Allelic Profiles	HBV n (%)	HCV n (%)	CG n (%)	*p*1	*p*2
*IL8*-251 A/T					
AA	17 (22.97)	20 (20)	54 (18)	0.1971	0.9044
AT	41 (55.40)	49 (49)	150 (50)		
TT	16 (21.62)	31 (31)	96 (32)		
A	0.51	0.45	0.43	0.3212	0.8867
T	0.49	0.55	0.57		

*p*1: HBV vs. CG; *p*2: HCV vs. CG; chi-squared test.

**Table 3:** Correlation of the *IL8*-251 A/T polymorphism with inflammatory activity and fibrosis score in chronic HBV and HCV carriers.

Genetic Profile	Inflammatory Activity			Fibrosis Score		
	0 to 1	2 to 3	*p*	0 to 2	3 to 4	*p*
	n (%)	n (%)		n (%)	n (%)	
*IL8*-251 A/T						
HBV						
AA	12 (19.7)	8 (50)	0.4212 *	11 (17.7)	6 (50)	1.000 *
AT	35 (57.4)	4 (25)		36 (58.1)	5 (41.7)	
TT	14 (22.9)	4 (25)		15 (24.2)	1 (8.3)	
A	0.48	0.63	0.0464 **	0.47	0.70	0.0016 **
T	0.52	0.37		0.53	0.30	
HCV			0.3724 **			0.5135 **
AA	11 (22)	6 (14.6)		15 (21.8)	5 (16.14)	
AT	27 (54)	20 (48.8)		35 (50.7)	14 (45.16)	
TT	12 (24)	15 (36.6)		19 (27.5)	12 (38.7)	
A	0.49	0.39	0.1998 **	0.47	0.39	0.3174 **
T	0.51	0.61		0.53	0.61	

Inflammatory activity: 0: absence of activity; 1: mild activity; 2: moderate activity; 3: intense activity. Fibrosis score: 0: absence of septa; 1: portal fibrosis without septa; 2: portal fibrosis with rare septa; 3: numerous septa but without cirrhosis; 4: cirrhosis; * G test; ** chi-squared test.

## Data Availability

The dataset of the current study will be made available from the corresponding author by reasonable request.
